# Perceived pay equity as a pathway from empowering leadership to innovation: the contingent role of psychological safety

**DOI:** 10.3389/fpsyg.2026.1752898

**Published:** 2026-05-12

**Authors:** Mingyeong Jeon, Meiling Yin

**Affiliations:** College of Business and Economics, Sejong University, Gwangjin-gu, Republic of Korea

**Keywords:** empowering leadership, innovation, moderated mediation, perceived pay equity, psychological safety, work environment

## Abstract

This study examines the impact of empowering leadership on employee innovation, with perceived pay equity as a key mediating mechanism. Drawing on data from a behavioral experiment and a survey, the results indicate that perceived pay equity significantly mediates the relationship between empowering leadership and innovation. Furthermore, moderated mediation analysis reveals that psychological safety moderates this relationship, such that the effect of empowering leadership is stronger among employees with lower levels of psychological safety. This finding suggests that psychologically less safe employees are more sensitive to leadership behaviors, highlighting the importance of fostering a supportive work environment alongside empowering leadership. Overall, this study contributes to the literature on leadership and innovation and offers practical implications for managers in organizations.

## Introduction

Does monetary incentive truly promote employees’ innovation? While financial rewards are commonly used to encourage innovative behavior, their effectiveness remains complicated. A key reason is that employees do not evaluate rewards in isolation but in comparison with others, making perceptions of pay fairness critical in engaging innovation (Buttner and Lowe, 2017a; Salimi and Della Torre, 2022). Drawing on equity theory (Adams, 1965), perceived inequity can lead to dissatisfaction and reduced motivation, ultimately hindering innovation. Despite substantial organizational investments in compensation systems (Scott et al., 2005), it remains unclear whether such investments effectively foster innovation when employees perceive unfairness. This raises an important yet underexplored question: what factors shape employees’ perceptions of pay equity, and how do these perception influence their innovative behavior?

Increasingly, leadership has been recognized as a critical factor in shaping employees’ motivation for innovative outcomes, with particular attention to empowering leadership, which involves granting employees autonomy and authority (Ford and Fottler, 1995; Vecchio et al., 2010). By enhancing employees’ participation in decision-making, access to information, and sense of control over work processes, empowering leadership increases transparency in organizational practices. Such transparency is crucial for how employees evaluate pay fairness, as it provides clearer reference points for social comparison. In contrast to more directive leadership approaches, empowering leadership thus plays a particularly critical role in shaping perceived pay equity. However, despite its relevance, research has largely overlooked how empowering leadership determines employees’ fairness perceptions as a mechanism linking leadership to innovation.

Extant research has primarily examined this issue along two separate lines: the relationship between empowering leadership and innovation, and the effects of pay fairness on employee outcomes such as performance (Tian and Wang, 2023; Burhan and Khan, 2024; Pett et al., 2025; Al-Ansi et al., 2015; Saythongkeo et al., 2022; Tang et al., 2025). Although both streams report positive effects, they have largely evolved in isolation, offering limited insight into how leadership and fairness perceptions play integrative process in shaping employee innovation. Critically, prior research has not conceptualized perceived pay equity as a psychological mechanism linking leadership to innovation. While some evidence suggests that leadership behaviors can enhance perceptions of compensation fairness (Bacha and Walker, 2013), and emerging studies hint at a relationship between empowering leadership and pay fairness (Rakic and Barjaktarovic Rakocevic, 2025; Qiao et al., 2025), these insights remain fragmented and lack systematic theoretical integration. Consequently, how and why empowering leadership influences innovation through perceived pay equity remains unclear, representing a key gap in the literature.

However, the effect of empowering leadership depends on how employees interpret and respond to leadership signals, suggesting that this mechanism may vary across psychological contexts. Psychological safety is a shared belief that it is safe to take interpersonal risks (Edmondson, 1999) and plays a critical role in shaping these interpretations within organizations. When psychological safety is low, employees may be more sensitive to leadership cues when interpreting organizational practices, which can amplify the influence of empowering leadership on perceived pay equity. Conversely, when psychological safety is high, employees may feel less dependent on such cues, potentially weakening this relationship. Accordingly, psychological safety is likely to serve as a key boundary condition influencing the strength of the psychological mechanism linking empowering leadership, perceived pay equity, and innovation.

Building on the proposed mechanism and its boundary condition, this study makes several contributions to organizational behavior literature, particularly research on leadership and employee innovation. First, it identifies perceived pay equity as a key psychological mechanism through which empowering leadership influences innovative behavior, thereby providing a deeper explanation of how leadership translates into innovation. Second, it bridges previously disconnected research streams by integrating leadership and pay fairness perspectives into a unified framework, offering a more comprehensive understanding of the joint role of leadership and fairness perceptions in shaping innovation (Tian and Wang, 2023; Hanafy et al., 2025; Pett et al., 2025). Third, it introduces psychological safety as a critical boundary condition, offering a novel perspective on when empowering leadership is more or less effective in shaping employees’ perceptions of pay equity. Fourth, it contributes methodologically by employing a multi-method approach: Study 1 applies scenario-based experiments to examine underlying mechanisms and establish causality, while Study 2 uses an employee survey to test the proposed relationships in a real-world organizational context. The combination of experimental methods and surveys enhances both internal and external validity, providing more robust and generalizable evidence. Finally, the study offers practical implications by highlighting that organizations should not only empower employees but also ensure fairness in pay and cultivate psychological safety to effectively foster innovation.

## Theoretical foundations and hypotheses development

### Empowering leadership and employees’ innovation

Scholars defined *empowering leadership* as leaders’ share of power, autonomy, and decision-making authority to their followers (Ford and Fottler, 1995; Vecchio et al., 2010). It is noted that empowering leadership positively affects the employees’ innovation, that is, the creation of novel ideas, services, products, processes, or management systems within an organization (Crossan and Apaydin, 2010). Many studies have shown that empowering leadership is closely related to innovative outcomes through mechanisms of diverse psychological factors such as empowerment and motivation (Abuzaid et al., 2024; Burhan and Khan, 2024; Hanafy et al., 2025; Pett et al., 2025).

Specifically, empowering leadership, which aligns closely with the self-determination view (Deci and Ryan, 1985), plays a critical role in fostering innovation by promoting self-directed behavior among employees. Leaders who adopt this leadership style can encourage employees to have independent thinking, proactive initiative, and the freedom to explore and experiment with new methods and ideas. This autonomy fosters an environment conducive to thinking outside the box, as employees feel encouraged to take ownership of their work and actively engage in problem-solving activities (Basu and Green, 1997; Amundsen and Martinsen, 2014, 2015; Elkhwesky et al., 2022), thereby facilitating the generation of innovation.

*H1*: *Higher levels of empowering leadership are positively associated with employees’ innovation.*

### Perceived pay equity as a mediating mechanism

*Perceived pay equity* refers to an individual’s perception that their compensation is fair relative to their contributions and compared with others, encompassing both satisfaction with pay and the fairness of its distribution (Goodman, 1974; Day, 2012; Fernandez and Quines, 2023). Beyond the structural design of compensation systems, their effectiveness largely depends on how employees interpret and evaluate these practices (De Spiegelaere et al., 2018; Amore and Failla, 2020). Drawing on equity theory (Adams, 1965), employees assess fairness by comparing their inputs and outcomes with those of others. When compensation is perceived as equitable, it can promote motivation and satisfaction; in contrast, perceived inequity tends to generate dissatisfaction and reduce effort. As a form of outcome-based organizational justice, perceived pay equity is particularly critical because it directly shapes motivational states that reinforce innovation-related behaviors.

Building on the importance of perceived pay equity, leadership plays a critical role in shaping how employees evaluate the fairness of compensation. Complementing equity theory, both social exchange theory and leader-member exchange (LMX) theory (Blau, 1964; Graen and Uhl-Bien, 1995) suggest that employees rely on leadership behavior as a key cue when forming fairness judgments. In this regard, empowering leadership by delegating autonomy and involving employees in decision-making signals recognition, trust, and value. From a social exchange perspective, such treatment is interpreted as fair and extracts positive reciprocal attitudes, while LMX theory further indicates that high-quality leader-member exchanges strengthen these fairness perceptions. Moreover, by increasing employees’ involvement in organizational processes, empowering leadership increases procedural fairness, which subsequently shapes evaluations of distributive outcomes such as pay equity. Consistent with this view, leadership that promotes transparency and participation has been shown to enhance employees’ evaluations of compensation fairness (Bacha and Walker, 2013). As a result, when employees perceive that they have a voice in reward-related processes, they are more likely to view compensation outcomes as fair, even in the presence of pay differences.

This perspective aligns with prior research showing that fair compensation can stimulate innovative behavior (Fung, 2009). Empowering leadership contributes to these outcomes by shaping pay equity perceptions through both relational and structural pathways. Relationally, it fosters trust and high quality LMX, while structurally, it promotes transparency and fairness in reward systems (Sparr and Sonnentag, 2008). In doing so, employees interpret leadership signals of recognition and trust as indicators of their value, which influences both pay evaluation and motivation to innovate (Grimpe et al., 2019). Consequently, employees who perceive pay equity report higher satisfaction with their compensation (Jawahar and Stone, 2011), which in turn boosts motivation and engagement, a key driver of innovative behavior (Antoni and Syrek, 2012; Ruiz-Palomino et al., 2013; Horoub and Zargar, 2022; Fernandez and Quines, 2023). Overall, empowering leadership fosters employee participation and enhances procedural fairness, which in turn stimulates perceptions of pay equity and motivates employees to engage in innovative behavior.

*H2*: *Perceived pay equity mediates the relationship between empowering leadership and employees’ innovation.*

### The moderating effect of psychological safety

Extending this logic, psychological safety shapes how employees rely on leadership cues when forming fairness evaluations. *Psychological safety* is a shared belief among employees that it is safe to take interpersonal risks within the organization (Edmondson, 1999). In the context of organizational behavior, psychological safety influences how employees interpret and respond to their work environment, particularly in situations involving uncertainty, evaluation, and social judgment (Edmondson, 2002). Employees rely on contextual cues to interpret leadership behaviors and organizational practices, and psychological safety constitutes an important condition that shapes this interpretive process (Blau, 1964; Graen and Uhl-Bien, 1995).

From a social exchange perspective, psychological safety alters employees’ reliance on relational cues, thereby conditioning how leadership behaviors are interpreted when forming fairness judgments. When psychological safety is low, employees experience greater uncertainty and rely more heavily on external cues when evaluating organizational practices. In such contexts, empowering leadership through autonomy, participation, and recognition serves as a salient cue that employees use to infer fairness in organizational practices. As a result, the positive relationship between empowering leadership and perceived pay equity is strengthened (Islam et al., 2024; Rakic and Barjaktarovic Rakocevic, 2025; Qiao et al., 2025).

In contrast, when psychological safety is high, employees feel safe and supported in their work environment. Under such conditions, they are less dependent on leadership cues to interpret organizational practices, and the influence of empowering leadership on perceived pay equity becomes less influential and may not be significant (Contu and Willmott, 2003). Psychological safety does not explain how empowering leadership affects perceived pay equity, but rather conditions the strength of this relationship. Thus, it functions as a boundary condition that strengthens or attenuates the effect of empowering leadership and perceived pay equity.

*H3*: *Psychological safety moderates the relationship between empowering leadership and perceived pay equity, such that the positive relationship is significant when psychological safety is low, but not significant when psychological safety is high.*

**Figure 1:**
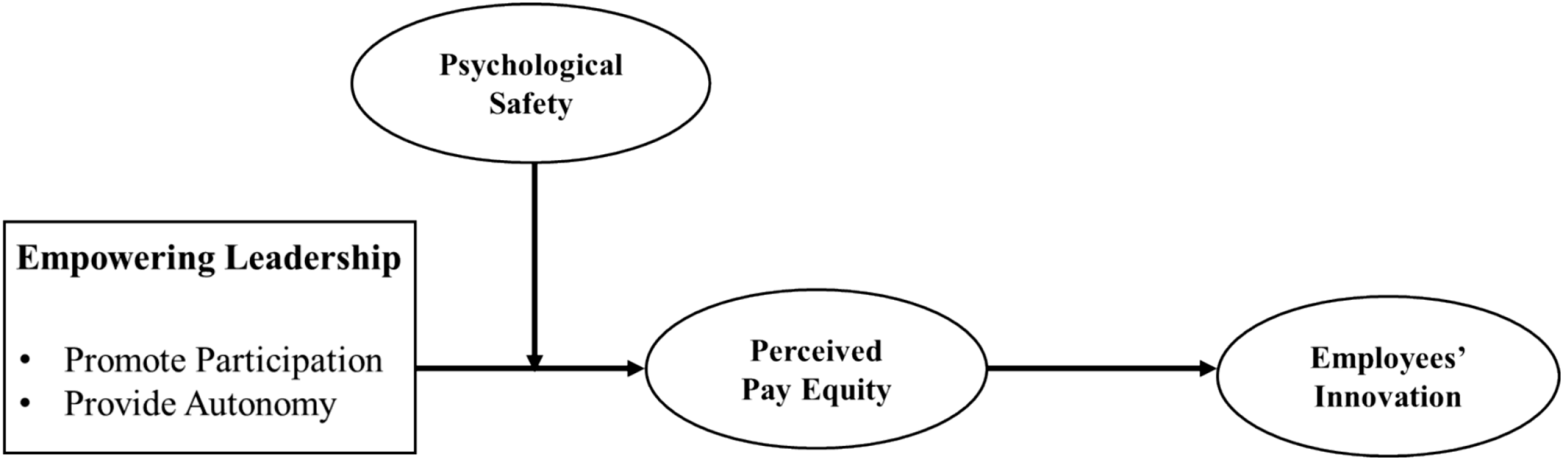
Research model.

The research model that we suggest is as follows (Figure 1). We propose that empowering leadership is positively related to employees’ innovation through perceived pay equity. Importantly, psychological safety moderates the relationship between empowering leadership and perceived pay equity.

## Study 1

### Sample and procedure

To examine the underlying mechanisms and establish causal relationships, we conducted a behavioral experiment to explore the mediating processes linking empowering leadership and employee innovation. Participants were recruited via Prolific from the United States and were required to have prior work experience. The final sample comprised 121 individuals (59 females, M_age_ = 40.67, SD = 10.99). Participants were randomly assigned to one of two conditions: a low level of empowering leadership condition (*n* = 60) or a high level of empowering leadership (*n* = 61).

We employed a between-participants design in which participants were randomly assigned to two different empowering leadership conditions. To manipulate empowering leadership, participants read a modified scenario adapted from Sun et al. (2022) and were instructed to imagine themselves working in the described environment. In the high empowering leadership condition, the leader encouraged participation in decision-making, consulted employees in developing work plans and strategies, granted autonomy, set clear yet flexible goals, fostered creativity, and provided developmental feedback. In contrast, in the low empowering leadership condition, the leader made unilateral decisions, limited participation and creativity, provided vague and inflexible goals, enforced strict adherence to rules, and focused feedback primarily on mistakes rather than development.

Following the scenario, participants completed several measures. The perceived pay equity was assessed using three items (Buttner and Lowe, 2017b; Cronbach’s alpha = 0.96), and innovation was measured with six items (Scott and Bruce, 1994; Anderson et al., 2014; Cronbach’s alpha = 0.98). A manipulation check was then conducted using a six-item empowering leadership scale (Ahearne et al., 2005; Zhang and Bartol, 2010; Cronbach’s alpha = 0.98), after which participants provided demographic information.

### Results

Participants in the high empowering leadership condition reported significantly higher perception of empowering leadership compared to those in the low condition (M_low_ = 2.18 [SD = 1.49], M_high_ = 5.66 [SD = 0.99]; *t* (1, 120) = 15.17, *p* < 0.001), confirming the effectiveness of manipulation.

Results indicated that participants in high level of empowering leadership condition showed significantly higher levels of perceived pay equity (M_low_ = 3.24 [SD = 1.36], M_high_ = 4.98 [SD = 1.27]; *t* (1, 120) = 7.24, *p* < 0.001) and innovation (M_low_ = 3.31 [SD = 1.70], M_high_ = 5.89 [SD = 0.85]; *t* (1, 120) = 10.64, *p* < 0.001) compared to those in low condition, thereby supporting H1.

We tested the proposed mediation using Hayes’ PROCESS Model 4, with empowering leadership (0 = low level of empowering leadership, 1 = high level of empowering leadership). The results revealed a significant indirect effect of perceived pay equity on the relationship between empowering leadership and innovation (*β* = 0.65, SE = 0.17; 95% CI = [0.33, 1.00]), supporting H2. These findings indicate that empowering leadership increases employee innovation, with perceived pay equity serving as a mediating mechanism.

This study supports our hypothesis that empowering leadership improves employees innovation (H1) and that this relationship is mediated by perceived pay equity (H2). However, several limitations should be acknowledged. First, although experimental designs allow for greater control by minimizing external influences, the use of manipulated scenarios may restrict external validity, as such conditions may not fully capture real-world organizational contexts. Second, as the sample consisted solely of employees from the United States, the generalizability of these findings to other cultural contexts, such as the Republic of Korea, which is characterized by a more collectivistic culture, remains uncertain. To address these limitations, the present research further examines whether cultural differences influence the relationships proposed in H1 and H2, and further verifies H3 regarding the moderating effect of perceived psychological safety.

## Study 2

### Sample and procedure

The sample selection of this study focused on the technology-intensive firms in the Republic of Korea, where require continuous innovation. Data were collected from companies located in Seoul and Gyeonggi-do using a questionnaire administered on a voluntary basis between May 2025 and November 2025. The questionnaire, originally developed in English, was translated into Korean using a rigorous forward–backward translation procedure to ensure linguistic and cultural equivalence. Two independent bilingual translators, fluent in both Korean and English, compared the back-translated and original versions to identify and resolve discrepancies. After minor revisions to enhance clarity and relevance for Korean employees, the final online survey was distributed via email to 282 participants.

Of the 252 returned questionnaires, 234 were deemed valid for analysis. The sample consisted of 110 (47.0%) women and 124 (53.0%) men. In terms of age distribution, 15 (6.4%) of respondents were under 28 years old, 187 (79.9%) were between 29 and 44 years old, and 32 (13.7%) were between 45 and 60 years old. Regarding organizational size, 39 (16.7%) of participants worked in firms with fewer than 50 employees, 30 (12.8%) in firms with 50–299 employees, 37 (15.8%) in firms with 300–999 employees, 35 (15.0%) in firms with 1,000–4,999 employees, and 93 (39.7%) in firms with 5,000 or more employees. With respect to job position, 32 (13.7%) were general staff, 65 (27.8%) were grassroots managers, 97 (41.5%) were middle managers, 23 (9.8%) were senior managers, and 17 (7.3%) were executives.

### Measures

The survey included measures of empowering leadership, perceived pay equity, employee innovation, and psychological safety. Empowering leadership was assessed with six items reflecting the degree of decision-making and work autonomy granted by participants’ managers (Ahearne et al., 2005; Zhang and Bartol, 2010). Perceived pay equity was measured using three items capturing participants’ evaluation of compensation fairness (Buttner and Lowe, 2017b). Employees innovation was assessed with six items reflecting innovative outcomes (Scott and Bruce, 1994; Anderson et al., 2014), and psychological safety was measured with three items assessing the extent to which participants felt safe to express themselves without fear of negative consequences (Edmondson, 1999).

Consistent with prior research, gender, age, organizational size, and job position were controlled for, given their potential association with empowering leadership and employees innovation (Zhang et al., 2024). All items were measured using a 7-point Likert scale, and the detailed items are reported in the Appendix.

### Results

#### Assessment of common method bias

Harman’s single-factor test was performed, and the unrotated factor accounted for 45.9% of the total variance, which is below the 50% threshold for all observed indicators; therefore, common method bias was not an issue. Furthermore, the four-factor model, including empowering leadership, perceived pay equity, innovation, safety demonstrated a significantly better fit (χ^2^/df = 2.944, SRMR = 0.051, CFI = 0.928, TLI = 0.914, *p* < 0.001) than the single-factor model (χ^2^/df = 11.133, TLI = 0.553, CFI = 0.606, RMSEA = 0.208, SRMR = 0.152). Therefore, common method bias does not appear to be a significant concern, and the data are suitable for further analysis.

#### Confirmatory factor analysis

**Figure 2:**
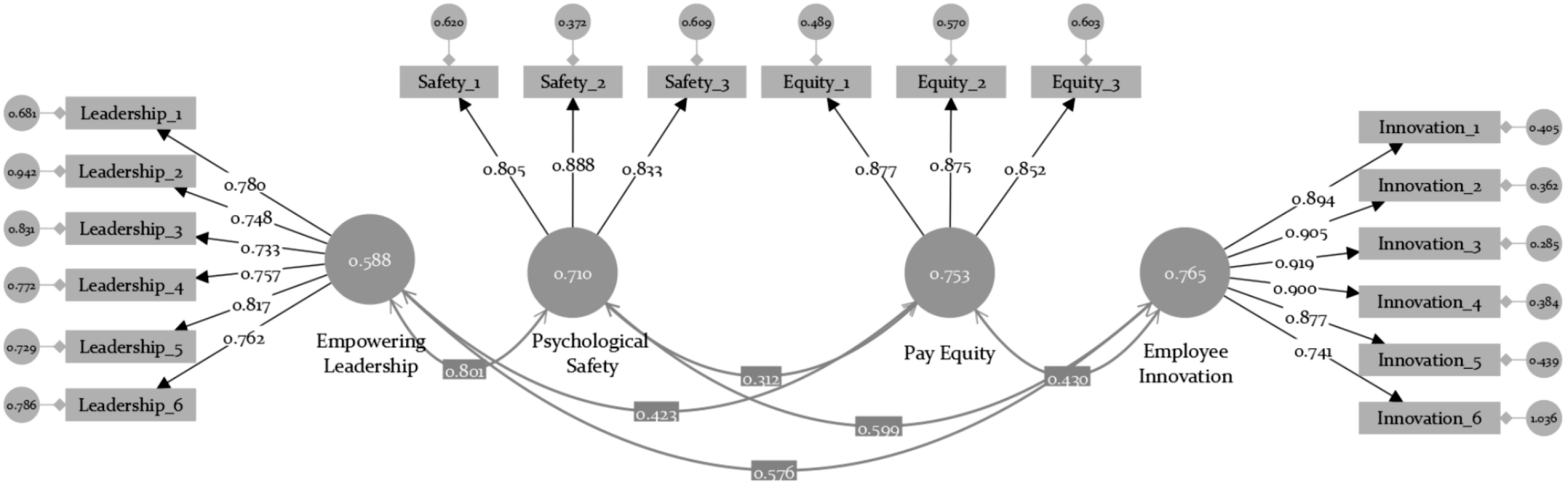
The results of the CFA. Model fit: χ^2^/df = 2.944, CFI = 0.928, GFI = 0.839, TLI = 0.914, SRMR = 0.051, RMSEA (0.091; LO90 = 0.081; HI90 = 0.102), *p* < 0.001.

The confirmatory factor analysis (CFA) was conducted using Smart PLS (Ver. 4) to find the structure of variables for each construct. Cronbach’s alpha values and composite reliability (CR) were above 0.8 (Table 1), indicating a good level of reliability. The Fornell-Larker criterion results confirmed that the square root of all AVE values was higher than the estimated correlation between the components of this study (Fornell and Larcker, 1981) (Table 1), thus demonstrating acceptable discriminant validity (Figure 2).

**Table 1:** Standardized factor loadings, AVE, CR, and discriminant validity.

Variable	Standard loading	Cronbach’s alpha	CR	AVE	Empowering leadership	Pay equity	Employee innovation	Psychological safety
Leadership_1	0.780	0.895	0.896	0.588	0.767			
Leadership_2	0.748
Leadership_3	0.733
Leadership_4	0.757
Leadership_5	0.817
Leadership_6	0.762
Equity_1	0.877	0.901	0.902	0.753	0.423	0.868		
Equity_2	0.875
Equity_3	0.852
Innovation_1	0.894	0.949	0.950	0.765	0.576	0.430	0.875	
Innovation_2	0.905
Innovation_3	0.919
Innovation_4	0.900
Innovation_5	0.877
Innovation_6	0.741
Safety_1	0.805	0.878	0.880	0.710	0.801	0.312	0.599	0.843
Safety_2	0.888
Safety_3	0.833

#### Descriptive analyses

The means, standard deviations (SD), and correlations for all variables are as follows. The empowering leadership (*M* = 4.75, SD = 1.13) has a significantly positive correlation with perceived pay equity (*M* = 3.98, SD = 1.37) (*r* = 0.375, *p* < 0.001) and employees innovation (M = 4.21, SD = 1.27) (*r* = 0.529, *p* < 0.001). Moreover, perceived pay equity has a significantly positive correlation with employees innovation (*r* = 0.395, *p* < 0.001). The variance inflation factor (VIF) analysis of the regression model showed that the highest VIF value for each variable remained under 3, below the accepted threshold of 10. These results indicate that there are no multicollinearity concerns in this study.

#### Hypothesis testing

To test the hypotheses, latent variables derived from the CFA were entered into a hierarchical regression model following procedures used in previous studies (Gao et al., 2025; Li J. et al., 2025; Li Y. et al., 2025). The results are reported in Table 2. After controlling for the variables of gender, age, organizational size, and job position, Model 6 shows that empowering leadership was positively related to employees innovation (*β* = 0.543, *p* < 0.001), providing support for H1.

**Table 2:** Linear regression analysis model.

Variable	Pay equity				Employees innovation			
	Model 1	Model 2	Model 3	Model 4	Model 5	Model 6	Model 7	Model 8
Gender	−0.342	−0.223	−0.220	−0.267	−0.481**	−0.309*	−0.377*	−0.270
Age	−0.010	−0.007	−0.008	−0.003	−0.016	−0.011	−0.013	−0.010
Lg (number of employees)	0.398***	0.349***	0.352***	0.321**	0.239*	0.168*	0.118	0.106
Grassroots manager	0.385	0.107	0.118	0.020	0.916*	0.516	0.799*	0.497
Middle manager	0.522	0.286	0.291	0.141	0.692	0.352	0.533	0.301
Senior manager	0.265	0.027	0.031	−0.010	0.311	−0.033	0.230	−0.037
Executive manager	−0.187	−0.238	−0.233	−0.302	−0.012	−0.085	0.044	−0.043
Empowering leadership		0.378***	0.343**	0.349**		0.543***		0.476***
Pay equity							0.304***	0.179**
Safety			0.045	0.007				
Empowering leadership*Safety				−0.142*				
*R* ^2^	0.129	0.216	0.217	0.237	0.118	0.327	0.212	0.357
Adjusted R^2^	0.102	0.188	0.186	0.202	0.09	0.303	0.184	0.331
▷R^2^	0.129	0.087	0.001	0.020	0.118	0.210	0.094	0.029
F	4.800***	7.759***	6.897***	6.909***	4.306***	13.685***	7.553***	13.789***

*p < 0.05; **p < 0.01; ***p < 0.001.

Empowering leadership is positively associated with perceived pay equity (*β* = 0.378, *p* < 0.001; see Model 2), which in turn was positively related to employee innovation (*β* = 0.304, *p* < 0.001; see Model 7). When perceived pay equity was entered as a mediator, it remains a significant predictor of employee innovation (*β* = 0.179, *p* < 0.01), while the direct effect of empowering leadership also remains significant (*β* = 0.476, *p* < 0.001; Model 8). This pattern of results suggests a mediating effect of perceived pay equity, supporting H2.

**Figure 3:**
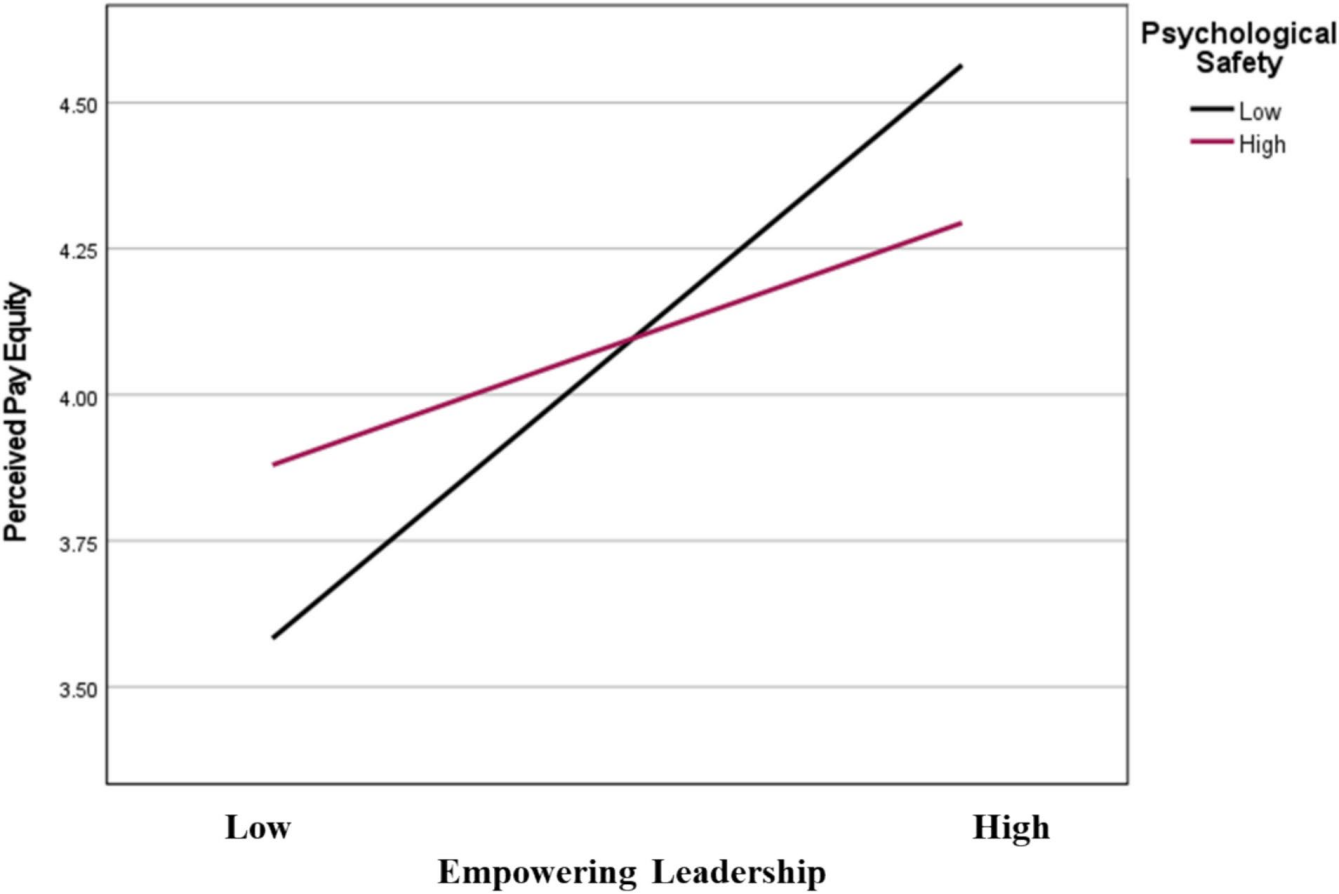
The interaction effect between empowering leadership and psychological safety on perceived pay equity.

To reduce multicollinearity when testing the moderating effect, empowering leadership and psychological safety were mean-centered, and then multiplied to construct the interaction term, and subsequently, hierarchical regression was used to verify the interaction effects. The interaction effect of empowering leadership and psychological safety on perceived pay equity was significant (*β* = −0.142, *p* < 0.05), as shown in Model 4. To further verify this relationship, different levels of psychological safety (mean-centered) were explored to examine the impact of empowering leadership on employees’ perceived pay equity. As shown in Figure 3, when psychological safety is low (*β* = 0.436, *p* < 0.001, 95% CI = [0.22, 0.66]), the slope of the positive relationship between empowering leadership and employees’ perceived pay equity is significant, but this relationship is insignificant in groups with high psychological safety (*β* = 0.184, *p* > 0.10, 95% CI = [−0.06, 0.43]), indicating that the positive effect of empowering leadership on perceived pay equity is significant among employees with lower levels of psychological safety, hence, H3 was supported.

Finally, to test whether perceived pay equity explained the effect of empowering leadership and psychological safety on employees’ innovation, we conducted a moderated mediation analysis using model 7 by Hayes PROCESS MACRO (Hayes and Preacher, 2010). The results revealed a significant index of moderated mediation (*β* = −0.024, SE = 0.011, 95% CI = [−0.05, −0.01]). The indirect effect through perceived pay equity was significant in groups with low psychological safety (*β* = 0.112, SE = 0.036, 95% CI = [0.05, 0.19]) but not in groups with high psychological safety (*β* = 0.053, SE = 0.035, 95% CI = [−0.01, 0.13]).

## Discussion and conclusion

This study examined how empowering leadership influences employee innovation by identifying perceived pay equity as a mediating mechanism and psychological safety as a boundary condition. The findings provide several insights that extend and refine existing research and offer a more nuanced understanding of leadership-driven innovation.

First, the results demonstrate that perceived pay equity plays a key mediating role in the relationship between empowering leadership and innovation. Prior research has largely examined the effects of leadership and pay fairness on innovation separately (Ahearne et al., 2005; Buttner and Lowe, 2017a, 2017b; Amore and Failla, 2020), focusing either on the direct link between leadership and innovation or on the impact of pay fairness on employee outcomes. Our study addresses this gap by identifying perceived pay equity as a psychological mechanism through which empowering leadership translates into innovative outcomes.

Specifically, empowering leadership shapes how employees interpret organizational practices, leading them to perceive compensation systems as fairer (Bacha and Walker, 2013). These fairness perceptions, in turn, enhance employees’ motivation to engage in innovative behavior. While prior research has primarily examined the direct effects of empowering leadership on innovation (Zhang and Bartol, 2010; Amundsen and Martinsen, 2014; Kim and Yoon, 2025), our findings provide a more comprehensive, process-oriented explanation by identifying employees’ subjective evaluations of fairness as a key underlying mechanism. This integrative perspective connects previously fragmented research streams on leadership, organizational justice, and innovation. Consistent with Kim and Beehr (2021), who demonstrate that empowering leadership fosters self-directed and proactive behaviors, our results further extend this view by highlighting perceived pay equity as a critical psychological pathway linking empowering leadership to employee innovation.

Second, this study contributes to the fairness and compensation literature by demonstrating that perceived pay equity is not only an outcome of organizational systems but also shaped by leadership. Previous studies have treated pay fairness primarily as a predictor of employee attitudes and behaviors (Buttner and Lowe, 2017a, 2017b), with limited attention to its antecedents. Our findings extend this perspective by showing that empowering leadership significantly influences how employees interpret compensation fairness, suggesting that fairness perceptions are not purely structural but socially constructed through leadership interactions. Supporting this argument, Fung (2009) and Ederer and Manso (2013) emphasize that the link between pay and innovation depends on employees’ interpretations of fairness and incentives, reinforcing the role of leadership in shaping these perceptions. In doing so, the study bridges the gap between leadership and compensation research and provides a more dynamic understanding of how fairness perceptions emerge within organizational contexts.

Third, our findings provide new insights into the role of psychological safety as a contextual boundary condition in shaping the impact of empowering leadership on perceived pay equity. Consistent with expectations, psychological safety moderates this relationship: the positive effect of empowering leadership on perceived pay equity is stronger when psychological safety is low. This suggests that in environments where employees feel less secure, they rely more heavily on leadership cues to interpret organizational practices and assess fairness. In contrast, when psychological safety is high, employees already feel supported and are less dependent on these leadership signals, reducing the incremental influence of empowering leadership. This aligns with prior research highlighting psychological safety as a key driver of knowledge sharing and open communication, which are critical for innovation (Jeon, 2024). Moreover, Du and Jin (2025) show that employees’ perceptions of risk in expressing themselves shape how leadership behaviors are interpreted, reinforcing our argument that psychological safety does not simply result from leadership but functions as a boundary condition that conditions employees’ fairness evaluations. Together, these findings indicate that empowering leadership is most influential in enhancing perceived pay equity and ultimately innovation under conditions of lower psychological safety, highlighting the contingent nature of leadership effectiveness in organizational contexts.

Finally, the study’s multi-method approach strengthens the robustness of findings. Study 1 establishes causal relationships through controlled scenario-based experiments while Study 2 captures employees’ perceptions in real organizational contexts, enhancing ecological validity. The convergence of findings across these designs provides triangulated evidence and supports the proposed moderated mediation model.

In conclusion, the present study advances the leadership and innovation literature by highlighting perceived pay equity as a key psychological mechanism linking empowering leadership to employee innovation, and by demonstrating that psychological safety conditions this process. These insights underscore the importance of fostering fairness perceptions and considering contextual factors to effectively promote innovative behaviors in organizations.

### Managerial implications

Although incentive systems play a critical role in motivating employees and encouraging innovation, our findings suggest that perceived pay equity is even more influential. Employees naturally compare their rewards with those of others, meaning that even well-designed incentive systems may fail to motivate innovation if employees perceive inequity. Therefore, organizations should not only focus on increasing incentives but also ensure that employees perceive compensation as fair.

To operationalize this, organizations should implement transparent and structured compensation practices. Managers should clearly communicate how pay and incentives are determined, specifying performance criteria, evaluation metrics, and reward allocation rules. Examples include standardized performance scorecards, published compensation ranges for different roles, and written explanations for bonus decisions. Regular compensation reviews and anonymous surveys can help monitor fairness perceptions and identify discrepancies early. Peer benchmarking systems can also provide employees with context on how their rewards compare to team or industry standards, reducing uncertainty and speculation.

While empowering leadership is widely encouraged, its effectiveness depends on whether it reinforces employees’ perceptions of fairness. Managers should engage in fairness-signaling behaviors, such as involving employees in decisions related to rewards, applying consistent performance standards, and documenting reward decisions. These behaviors can be embedded into leadership evaluation systems, where managers are assessed not only on team performance but also on perceived fairness and transparency. Leadership training programs should develop skills in transparent communication, unbiased evaluation, and participative decision-making, including simulations on conducting fair performance appraisals and delivering compensation feedback.

Finally, psychological safety determines when empowering leadership is most effective. Our findings indicate that empowering leadership has a stronger impact on perceived pay equity and innovation when psychological safety is low, as employees rely more on leadership cues in less supportive environments. Practically, this suggests that empowering leadership is particularly valuable in teams where employees are less comfortable speaking up. In such contexts, leaders should actively involve employees in decision-making, clearly communicate how compensation decisions are determined, and provide transparent and consistent feedback to reinforce fairness perceptions and encourage innovation. Organizations should continue to foster psychological safety through open communication and supportive practices, while recognizing that empowering leadership is especially critical in less supportive environments.

### Limitations and direction for future study

Our study findings should be interpreted in light of several limitations. First, due to design constraints in study 1, the moderating role of psychological safety was not experimentally manipulated. Future studies could directly manipulate psychological safety to more rigorously examine its moderating effect and to establish stronger causal evidence regarding how contextual conditions shape the relationship between empowering leadership and perceived pay equity.

Second, study 2 relied on a cross-sectional, self-reported design, which raises concerns regarding common method bias and limits causal inference. In particular, the mediation results should be interpreted with caution, as the temporal ordering among empowering leadership, perceived pay equity, and innovation cannot be firmly established. Future studies could employ longitudinal or multi-source designs to better capture the dynamic relationships among these variables and strengthen causal interpretations.

Furthermore, while this study examined innovation at the individual level, focusing on employees’ innovative behaviors, it does not capture broader collective dynamics. Future research could extend this approach to the team or organizational level, investigating how shared perceptions of pay equity and leadership styles influence collective innovation outcomes. Addressing these limitations would provide a more comprehensive understanding of how leadership and perceived pay equity jointly influence employee innovation.

## Data Availability

The original contributions presented in the study are included in the article/supplementary material, further inquiries can be directed to the corresponding author.

## Appendix: all measurement items

**Table:**

Variables	Items	
Empowering Leadership (Ahearne et al., 2005; Zhang and Bartol, 2010)	1	My manager makes many decisions together with me.
	2	My manager often consults me on strategic decisions.
	3	My manager solicits my opinion on decisions that may affect me.
	4	My manager allows me to do my job my way.
	5	My manager makes it more efficient for me to do my job by keeping the rules and regulations simple.
	6	My manager allows me to make important decisions quickly to satisfy customer needs.
Perceived pay equity (Buttner and Lowe, 2017b)	1	The pay for my job is excellent.
	2	I am satisfied with my current pay.
	3	The earnings on my job in comparison to others doing the same type of work that I do are fair.
Employees’ innovation (Scott and Bruce, 1994; Anderson et al., 2014)	1	I regularly come up with new and original ideas.
	2	I actively contribute creative solutions to problems.
	3	I often explore new ways of doing things in our work.
	4	I am effective at turning new ideas into practical actions.
	5	I not only come up with innovative ideas, but also implement them.
	6	Over the past 6 months, I have introduced at least one important new idea or method in my tasks or when doing things.
Perceived psychological safety (Edmondson, 1999)	1	I feel comfortable sharing my ideas and concerns with all team members, regardless of their status.
	2	I feel free to question decisions made by others, regardless of their status.
	3	I feel safe admitting when I do not understand something.
